# Obesity Prevalence and Associated Factors Among Users of Specialized Community Mental Health Services: An Observational Study

**DOI:** 10.1111/inm.70279

**Published:** 2026-06-10

**Authors:** Thaiane Bruna da Cruz Diomedece, Bruno da Silva Marinho, Ana Laura Salomé Lourencetti, Mark Costa, Maria O'Connell, Chyrell Bellamy, Carlos Alberto dos Santos Treichel

**Affiliations:** ^1^ Department of Maternal‐Child and Psychiatric Nursing, School of Nursing University of São Paulo São Paulo Brazil; ^2^ Department of Psychiatry Yale University New Haven Connecticut USA

**Keywords:** community mental health services, delivery of health care, mental health, obesity, physical health

## Abstract

In Brazil, approximately 6.9 million people live with severe, mild, or moderate mental disorders, requiring different levels of care. Psychosocial Care Centres play a fundamental role in providing specialized care to this population, promoting welcoming practices, continuous treatment, and social reintegration within the Psychosocial Care Network. Although effective in reducing crises and hospitalizations, challenges remain regarding the maintenance of users' physical health. This observational and analytical study aimed to identify the prevalence of obesity (defined as a body mass index ≥ 30 kg/m^2^) and its associated factors among 358 adults receiving care in three specialized mental health services in a medium‐sized municipality. A structured, content‐validated questionnaire collected sociodemographic, clinical, behavioural and service‐related information. Weight, height and waist circumference were measured following international standards. The prevalence of obesity was 32.1%, and an additional 32.7% were overweight, totaling 64.8% with excess weight. Lower prevalence of obesity was observed among users of services focused on the treatment of substance use disorders. Higher prevalence was identified among women, individuals who self‐identified as Black, people with hypertension, and those with poorer self‐rated health. Smoking was associated with lower obesity prevalence. It is concluded that obesity is highly prevalent among users of community mental health services, highlighting the need for integrated strategies that incorporate physical health as an essential component of mental health care.

## 1 Introduction

Obesity is a significant and growing public health issue worldwide, and individuals living with mental disorders are disproportionately affected due to clinical, behavioural, and social vulnerabilities (Islam et al. 2024). International evidence has consistently demonstrated strong associations between mental disorders, psychotropic medication use, and metabolic complications (Sepúlveda‐Lizcano et al. 2023). Despite this well‐established global concern, data from low and middle‐income countries (LMICs) remain limited (Afzal et al. 2021). In upper‐middle‐income countries such as Brazil, evidence is still scarce, particularly regarding the prevalence of obesity among individuals receiving community‐based mental health care (Jardim et al. 2017).

While Brazil has undertaken significant psychiatric reforms and expanded community mental health services, its health system still struggles to integrate physical and mental health care (Rathod et al. 2017; Dalgalarrondo et al. 2023). A critical symptom of this fragmented care is the absence of robust data on prevalent comorbidities such as obesity. National data on obesity within specialized mental health settings remain scarce, fragmented, and insufficient to guide policies or interventions (Jardim et al. 2017; Afzal et al. 2021). Strengthening the evidence base in this context is essential for improving health care in Brazil and advancing global efforts to tackle how mental health systems can better address physical health disparities (Rajan and Menon 2017).

Therefore, the aim of this study was to identify the prevalence of obesity and the factors associated with it among users of specialized mental health services in a municipality of São Paulo, Brazil. By addressing a significant gap in the literature from a Brazilian perspective, this research may inform both national and international strategies aimed at improving the comprehensiveness of community mental health care.

## 2 Background

Worldwide, individuals with severe mental disorders experience marked physical health disparities, including higher rates of chronic diseases and premature mortality (Dragioti et al. 2023). Health systems across different regions face persistent challenges in integrating physical and mental health care, especially within community‐based service models (Monaghan and Cos 2021). Psychotropic medication‐related adverse effects, insufficient health promotion activities, and fragmentation between levels of care contribute to the deterioration of physical health among mental health service users (Taipale et al. 2020; Fiorillo and Sartorius 2021).

Brazil provides an illustrative example of these challenges. Approximately 6.9 million Brazilians live with severe mental disorders requiring continuous care, while an additional 30.2 million experience mild or moderate conditions requiring occasional support (WHO 2017). Established in 2011, the Psychosocial Care Network (Rede de Atenção Psicossocial—RAPS) organizes mental health services across all levels of care in the country, ensuring continuity, including crisis and emergency responses. It encompasses primary care, community‐based services, specialized outpatient care, and hospital services. Within this network, Psychosocial Care Centres (Centros de Atenção Psicossocial—CAPS) are the main community‐based services, providing continuous, territorially based care for individuals with severe mental disorders, supporting deinstitutionalization and reducing hospitalizations, with different modalities for adults, substance use disorders, and children and adolescents (Onocko‐Campos et al. 2018).

Studies conducted internationally and in Brazil have demonstrated the effectiveness of community mental health services in reducing psychiatric crises, decreasing hospitalizations, and promoting functional recovery and quality of life (Tomasi et al. 2010; Rodrigues et al. 2016; Taban et al. 2024; Černe Kolarič et al. 2024). However, the physical health of service users remains a critical concern across different contexts (Franzmann et al. 2018; Dragioti et al. 2023; Rigo and Treichel 2024). Obesity, in particular, is highly prevalent among people with mental disorders due to factors such as psychotropic‐induced weight gain, reduced physical activity, and inadequate dietary patterns (Rajan and Menon 2017; Jardim et al. 2017; Taipale et al. 2020; Afzal et al. 2021).

Despite robust international literature, research on obesity in community mental health settings in Upper‐Middle‐Income Countries, including Brazil, remains limited (Afzal et al. 2021). The scarcity of context‐specific data restricts the development of evidence‐informed public policies and targeted interventions, underscoring the need for studies that advance understanding of physical health inequities among people with mental disorders in diverse health system contexts.

This study aims to examine the prevalence of obesity and the factors associated with it among individuals receiving care in specialized mental health services in a municipality in São Paulo, Brazil. To address this objective, the following sub‐aims will be targeted:

- To determine the Body Mass Index (BMI) of users receiving care in specialized mental health services in this municipality.
- To identify sociodemographic, clinical, and service‐related factors associated with obesity in this population.

## 3 Methods

### 3.1 Study Setting and Sampling

This observational analytical study was conducted in Itatiba, a medium‐sized municipality in the state of São Paulo, Brazil, with an estimated population of 122 581 inhabitants (IBGE 2023). The local health system includes 19 Primary Health Care (PHC) units, hospital and emergency services, and three specialized mental health services: A Psychosocial Care Centre II (CAPS II), a Psychosocial Care Centre for alcohol and drug use (CAPS AD), and a mental health outpatient clinic. Approximately 1958 individuals receive care in these services, according to previous survey data (Treichel and Campos 2022).

The target population comprised users enrolled in three specialized mental health services within the municipality's Psychosocial Care Network (CAPS AD, CAPS II, and an outpatient clinic). A simple random sampling strategy was undertaken by the research team using lists generated from service medical records. The sample size was calculated using a proportion estimation formula, assuming a population of 2000 users, a hypothetical prevalence of 50% (to maximize sample size), a 95% confidence interval, and an additional 20% to account for potential losses, resulting in 387 users. Sampling was stratified by service. Eligible users were randomly selected, contacted by telephone, and invited to participate; all those who agreed were included, yielding a final sample of 358 participants.

### 3.2 Inclusion and Exclusion Criteria

The inclusion criterion was being registered with the mental health service for at least one month. Participants under 18 years of age were excluded. Those with a diagnosis of intellectual disability were also excluded, as this condition could limit their understanding of the questions.

### 3.3 Instrument and Measures

Data were collected using a structured questionnaire developed based on a literature review. Prior to data collection, the questionnaire underwent content validation through a series of workshops involving researchers, mental health professionals, and service users. The administration of the questionnaire took approximately 30 to 60 min per participant.

Independent variables included service type, sex, race/ethnicity, age, marital status, educational level, employment status, per capita income, diagnosis, time in service, tobacco use, alcohol use, physical activity, hypertension, diabetes, Primary Health Care (PHC) utilization in the previous six months, hospitalization in the previous year, community health worker visits, and self‐rated health.

Anthropometric measures, namely, weight, height, and waist circumference, were also collected following the World Health Organization standardization protocols (WHO 1995). Weight was measured using a calibrated digital scale with participants barefoot and wearing light clothing. Height was assessed using a stadiometer, with participants standing upright in the Frankfort plane. Waist circumference was measured with a non‐elastic tape placed midway between the last rib and the iliac crest.

Body Mass Index (BMI) was calculated as weight (kg) divided by height squared (m^2^) and classified according to WHO criteria for adults (WHO 1998): underweight (BMI < 18.5), normal weight (18.5–24.9), overweight (25.0–29.9), and obesity (≥ 30.0). Waist circumference was measured and classified according to the increased morbidity/mortality risk thresholds established by the WHO, with a cut‐off point of ≥ 80 cm for women and ≥ 94 cm for men (WHO 1995).

### 3.4 Data Collection

Data collection was performed in person at the mental health services where participants received care. Trained data collectors, health professionals, or students administered the questionnaires using free software installed on tablets. Collectors received standardized training and were supervised by the principal investigator throughout the study.

Participants were contacted by telephone, informed about the study, and invited to participate. Data collection appointments were scheduled on days when users were already expected to attend the service for consultations or activities. At the time of data collection, the informed consent form was read aloud by the data collector, and questions were clarified before participants signed the form in duplicate.

### 3.5 Data Analysis

Data were analysed using Stata 18 (StataCorp, College Station, Texas, USA). The prevalence of obesity and other outcomes was calculated both for the total sample and across strata defined by independent variables.

Associations between obesity and covariates were assessed using Poisson regression models with robust variance estimators, presented as crude and adjusted prevalence ratios. Confounders were selected via a forward stepwise approach, with variables showing a *p*‐value ≤ 0.20 considered for inclusion in the multivariable model (Maldonado and Greenland 1993).

No missing data were observed for the variables included in the analyses.

## 4 Results

### 4.1 Participant Characteristics

A total of 358 participants were included in the study. The mean age was 44.8 years (SD = 14.7). The majority were female (53.3%, *n* = 191) and self‐identified as White (56.7%, *n* = 203). Most participants lived with a partner (68.1%, *n* = 244), while 31.8% (*n* = 114) were single. Regarding education, 43.3% (*n* = 155) had completed high school, 23.4% (*n* = 84) had completed up to elementary school (up to 4th grade), and 20.6% (*n* = 74) had completed up to middle school (up to 8th grade), and 12.5% (*n* = 45) had completed a university degree. Only 36.8% (*n* = 132) reported paid employment. Per capita family income was up to half the minimum wage for 37.9% (*n* = 136), up to one minimum wage for 40.7% (*n* = 146), and above one minimum wage for 21.2% (*n* = 76).

Participants were recruited across three mental health services: 39.4% (*n* = 141) attended CAPS II, 31.3% (*n* = 112) the outpatient clinic, and 29.3% (*n* = 105) CAPS AD. The most frequent clinical diagnoses were substance use disorders (24.8%, *n* = 89) and anxiety disorders (24.3%, *n* = 87), followed by psychotic disorders (14.8%, *n* = 53), unipolar mood disorders (12.8%, *n* = 46), bipolar disorder (8.1%, *n* = 29), and other/unspecified conditions (15.0%, *n* = 54). Mental disorder diagnoses were grouped according to the ICD classification based on clinical records in patient charts.

Time in care varied: 26.6% (*n* = 94) had been in treatment for up to 6 months, 19.8% (*n* = 71) for up to 2 years, 22.3% (*n* = 80) for up to 5 years, 11.4% (*n* = 41) for up to 10 years, and 20.1% (*n* = 72) for more than 10 years.

Table 1 presents detailed sociodemographic and clinical characteristics of the study population.

**TABLE 1:** Sociodemographic profile of participants included in the study (*n* = 358). Itatiba‐SP, Brazil, 2025.

	*n*	%
Service		
Outpatient Clinic	112	31.3
CAPS II	141	39.4
CAPS AD	105	29.3
Sex		
Male	167	46.5
Female	191	53.3
Race/Ethnicity		
White	203	56.7
Brown (Mixed race)	110	30.7
Black	45	12.5
Age group		
18–30 years	69	19.2
31–40 years	80	22.3
41–50 years	81	22.6
51–60 years	67	18.7
61 years or older	61	17.0
Marital status		
Single	114	31.8
Living with a partner	244	68.1
Educational level		
Up to 4th grade	84	23.4
Up to 8th grade	74	20.6
Up to high school	155	43.3
Higher education	45	12.5
Paid employment		
Yes	132	36.8
No	226	63.1
Per capita family income		
Up to half the minimum wage	136	37.9
Up to one minimum wage	146	40.7
More than one minimum wage	76	21.2
Diagnosis		
Anxiety disorders	87	24.3
Unipolar mood disorders	46	12.8
Bipolar disorder	29	8.1
Psychotic disorders	53	14.8
Substance use–related disorders	89	24.8
Other or no assigned diagnosis	54	15.0
Time in service		
Up to 6 months	94	26.6
Up to 2 years	71	19.8
Up to 5 years	80	22.3
Up to 10 years	41	11.4
More than 10 years	72	20.1

### 4.2 Obesity Prevalence and Nutritional Outcomes

Based on the Body Mass Index classification, 32.1% (*n* = 115) of participants met criteria for obesity (BMI ≥ 30 kg/m^2^). An additional 32.7% (*n* = 117) were overweight (BMI 25.0–29.9 kg/m^2^), resulting in 64.8% (*n* = 232) of the sample presenting excess body weight. A total of 32.4% (*n* = 116) were within the normal range, and 2.8% (*n* = 10) were underweight.

Waist circumference measurements indicated that 65.6% (*n* = 235) of participants had values consistent with increased metabolic risk (≥ 80 cm for women and ≥ 94 cm for men).

Regarding anthropometric monitoring, 59.5% (*n* = 213) reported having had their weight and height measured by a health professional within the previous month, 20.4% (*n* = 73) within the last six months, 7.0% (*n* = 25) within a year, and 3.9% (*n* = 14) more than one year ago. Notably, 9.2% (*n* = 33) reported never having undergone anthropometric assessment by a health professional. Detailed monitoring data are presented in Table 2.

**TABLE 2:** Anthropometric indicators and self‐reported anthropometric monitoring by a health professional among study participants (*n* = 358). Itatiba‐SP, Brazil, 2025.

	*n*	%
Body Mass Index (BMI) (kg/m^2^)		
Underweight < 18.50	10	2.8
Normal weight 18.50–24.99	116	32.4
Overweight 25.00–29.99	117	32.7
Obesity ≥ 30	115	32.1
Waist circumference		
No increased morbidity/mortality risk	123	34.4
Increased morbidity/mortality risk (≥ 80 cm for women; ≥ 94 cm for men)	235	65.6
Time since last anthropometric assessment by a health professional		
Within 1 month	213	59.5
Within 6 months	73	20.4
Within 1 year	25	7.0
More than 1 year	14	3.9
Never had weight and height measured by a health professional	33	9.2

### 4.3 Factors Associated With Obesity

Table 3 presents crude and adjusted prevalence ratios for obesity according to sociodemographic, clinical, and service‐related characteristics.

**TABLE 3:** Prevalence and Prevalence Ratios of obesity among participants according to covariate strata (*n* = 358). Itatiba‐SP, Brazil, 2025.

	*n*	%	Crude PR (95% CI)	*p*	Adjusted PR (95% CI)^a^	*p*
Service						
Outpatient Clinic	112	40.18	1 (reference)	—	1 (reference)	—
CAPS II	141	37.59	0.98 (0.89–1.07)	0.675	1.00 (0.92–1.09)	0.909
CAPS AD	105	16.19	0.82 (0.75–0.90)	< 0.001	0.90 (0.81–0.99)	0.033
Sex						
Male	167	23.35	1 (reference)	—	1 (reference)	—
Female	191	39.79	1.13 (1.05–1.21)	0.001	1.08 (1.01–1.16)	0.020
Race/Ethnicity						
White	203	31.03	1 (reference)	—	1 (reference)	—
Brown (Mixed race)	110	28.18	0.97 (0.90–1.06)	0.598	0.99 (0.92–1.07)	0.932
Black	45	46.67	1.11 (1.00–1.25)	0.046	1.12 (1.00–1.25)	0.036
Age group						
18–30 years	69	30.43	1 (reference)	—	1 (reference)	—
31–40 years	80	27.50	0.97 (0.87–1.09)	0.694	0.97 (0.88–1.08)	0.687
41–50 years	81	33.33	1.02 (0.91–1.14)	0.704	0.99 (0.89–1.11)	0.975
51–60 years	67	40.30	1.07 (0.95–1.21)	0.227	1.01 (0.90–1.13)	0.791
≥ 61 years	61	29.51	0.99 (0.87–1.12)	0.908	0.90 (0.80–1.02)	0.103
Marital status						
Single	114	31.56	1 (reference)	—	1 (reference)	—
Living with a partner	244	33.33	1.01 (0.96–1.09)	0.738	0.96 (0.89–1.04)	0.426
Educational level						
Up to 4th grade	84	36.90	1 (reference)	—	1 (reference)	—
Up to 8th grade	74	36.49	0.99 (0.89–1.11)	0.957	0.99 (0.89–1.10)	0.941
Up to high school	155	29.68	0.94 (0.86–1.04)	0.257	0.97 (0.89–1.06)	0.603
Higher education	45	24.44	0.90 (0.80–1.03)	0.138	0.92 (0.81–1.04)	0.223
Paid employment						
Yes	132	25.76	1 (reference)	—	1 (reference)	—
No	226	35.84	1.08 (1.00–1.16)	0.044	1.05 (0.97–1.13)	0.156
Per capita family income						
Up to half the minimum wage	136	34.56	1 (reference)	—	1 (reference)	—
Up to one minimum wage	146	32.88	0.98 (0.90–1.07)	0.766	0.99 (0.91–1.07)	0.841
More than one minimum wage	76	26.32	0.93 (0.85–1.03)	0.208	0.91 (0.83–1.00)	0.076
Diagnosis						
Anxiety disorders	87	37.93	1 (reference)	—	1 (reference)	—
Unipolar mood disorders	46	43.48	1.04 (0.91–1.17)	0.534	1.09 (0.97–1.23)	0.126
Bipolar disorder	29	41.38	1.05 (0.88–1.18)	0.742	1.03 (0.90–1.19)	0.604
Psychotic disorders	53	33.96	0.97 (0.86–1.09)	0.635	1.00 (0.89–1.13)	0.886
Substance use–related disorders	89	19.48	0.82 (0.74–0.90)	< 0.001	0.89 (0.81–0.99)	0.043
Other or no assigned diagnosis	54	37.04	0.99 (0.88–1.11)	0.915	1.02 (0.91–1.15)	0.692
Smoking						
No	228	40.79	1 (reference)	—	1 (reference)	—
Yes	130	16.92	0.83 (0.77–0.89)	< 0.001	0.84 (0.79–0.91)	< 0.001
Alcohol use						
No	272	33.82	1 (reference)	—	1 (reference)	—
Yes	86	26.74	0.94 (0.86–1.03)	0.210	1.00 (0.92–1.09)	0.873
Physical activity						
Never	193	32.64	1 (reference)	—	1 (reference)	—
Occasionally	82	39.02	1.04 (0.95–1.14)	0.311	1.06 (0.97–1.15)	0.184
≥ 3 times/week	83	24.10	0.93 (0.85–1.02)	0.145	0.94 (0.86–1.02)	0.193
Hypertension						
No	254	25.59	1 (reference)	—	1 (reference)	—
Yes	104	48.08	1.17 (1.09–1.27)	< 0.001	1.14 (1.05–1.23)	0.001
Diabetes Mellitus						
No	306	30.39	1 (reference)	—	1 (reference)	—
Yes	52	42.31	1.09 (0.98–1.20)	0.094	1.00 (0.90–1.12)	0.879
Use of PHC unit in the past 6 months						
Did not use	113	27.43	1 (reference)	—	1 (reference)	—
Used	245	34.29	1.05 (0.97–1.13)	0.190	0.99 (0.92–1.07)	0.960
Hospitalization in the past year						
Not hospitalized	288	33.33	1 (reference)	—	1 (reference)	—
Hospitalized	70	27.14	0.95 (0.87–1.04)	0.309	0.96 (0.88–1.05)	0.440
Visits from CHW						
No	211	31.28	1 (reference)	—	1 (reference)	—
Yes	147	33.33	1.01 (0.94–1.09)	0.683	1.01 (0.94–1.08)	0.747
Self‐rated health						
Excellent or very good	60	16.67	1 (reference)	—	1 (reference)	—
Good	228	34.21	1.15 (1.04–1.26)	0.003	1.13 (1.13–1.24)	0.006
Poor or very poor	70	38.57	1.18 (1.05–1.33)	0.004	1.12 (1.00–1.25)	0.045

Abbreviations: CHW, community health worker; PHC, primary health care.

^a^ Adjusted for smoking and hypertension.

In the adjusted Poisson regression analysis with robust variance, several variables were significantly associated with obesity. Participants attending CAPS AD had a lower prevalence of obesity compared with those treated in the outpatient clinic (PR = 0.90; 95% CI: 0.81–0.99; *p* = 0.033), and individuals diagnosed with substance use disorders also presented a reduced prevalence when compared with participants with anxiety disorders (PR = 0.89; 95% CI: 0.81–0.99; *p* = 0.043). Regarding sociodemographic characteristics, female participants had a higher prevalence of obesity than males (PR = 1.08; 95% CI: 1.01–1.16; *p* = 0.020), and participants who self‐identified as Black had a higher prevalence compared with those identifying as White (PR = 1.12; 95% CI: 1.00–1.25; *p* = 0.036).

Behavioural and clinical factors also demonstrated significant associations. Smoking was negatively associated with obesity, with smokers showing a lower prevalence of the outcome compared with non‐smokers (PR = 0.84; 95% CI: 0.79–0.91; *p* < 0.001). Conversely, participants diagnosed with hypertension presented a higher prevalence of obesity than those without the condition (PR = 1.14; 95% CI: 1.05–1.23; *p* = 0.001). Finally, self‐rated health was associated with obesity: participants who rated their health as good (PR = 1.13; 95% CI: 1.13–1.24; *p* = 0.006) or poor/very poor (PR = 1.12; 95% CI: 1.00–1.25; *p* = 0.045) had a higher prevalence of the outcome compared with those who rated their health as excellent or very good.

## 5 Discussion

This study reveals a substantial burden of excess weight among users of specialized mental health services: 64.8% of participants were affected, with 32.1% classified as obese and an additional 32.7% as overweight. Our results are consistent with international evidence indicating that people with severe mental illness are significantly more likely to live with obesity than the general population. A recent meta‐analysis encompassing WHO member states reported a pooled overweight/obesity prevalence of 37% (Islam et al. 2024). In Brazil, although population‐based data indicate an increase in obesity from 11.1% in 2006 to 19.8% in 2019 (Lima et al. 2024), studies focusing on individuals with mental disorders also report high prevalence rates. For example, research conducted in southern Brazil found a prevalence of 34.8% for overweight and 21% for obesity among people with mental disorders (Jardim et al. 2017).

Another large meta‐analysis reported an obesity prevalence of 25.9% among individuals with severe mental illness and a combined overweight/obesity prevalence of 60.1% (Afzal et al. 2021). While the pooled obesity prevalence in high‐income settings tends to be lower than the prevalence found in this study, contextual factors such as socioeconomic vulnerability, racial inequities, food environments, medication profiles, and limited access to preventive care may contribute to the higher burden observed in Brazilian psychosocial care settings.

The elevated prevalence of obesity identified here aligns with previous Brazilian studies. For instance, Jardim et al. (2017) found rates of 34.8% for overweight and 21% for obesity among people with mental disorders in southern Brazil. However, the proportion of individuals with obesity in the present study (32.1%) is markedly higher, reinforcing the increasing epidemiological relevance of metabolic disorders in mental health populations and highlighting the unique vulnerabilities faced by users of the Brazilian Psychosocial Care Network.

Waist circumference findings further underscore the magnitude of metabolic risk, as 65.6% of participants demonstrated values compatible with increased morbidity and mortality risk. Central adiposity is a particularly relevant indicator among people with mental disorders (Lv et al. 2025) due to the complex interplay between psychotropic medication effects, lifestyle factors, and socioeconomic determinants (Rajan and Menon 2017; Jardim et al. 2017; Taipale et al. 2020; Afzal et al. 2021). However, international studies (Batscha et al. 2017; Mitchell et al. 2012) have shown that its assessment is often underutilized in metabolic monitoring among individuals with mental disorders, and that although crucial, the collection of waist circumference data in this population may face barriers related to the physical proximity required for measurement. In the Brazilian context, this challenge may be further exacerbated by the high demand at the specialized care level and the limited integration between services, which contributes to the fragmentation of care between mental and physical health (Amaral et al. 2018).

Regarding anthropometric monitoring, the study identified significant gaps in routine assessment. Although 59.5% of participants had their weight and height measured in the month preceding data collection, nearly one‐third reported assessments occurring only within the past year, and 9.2% reported never having been measured by a health professional. Comparatively, few studies report such detailed data on anthropometric monitoring among mental health service users, particularly in community‐based settings. The limited monitoring observed here suggests a missed opportunity for early identification of risk and for integrated care practices that align mental health and general health needs. This reinforces the critical role of nurses, who are pivotal in conducting screening, coordinating care, and promoting metabolic health monitoring in psychosocial care services (Ward et al. 2018). Nursing professionals are often the primary providers responsible for ongoing contact with users, positioning them uniquely to implement systematic assessments and strengthen continuity of care (Fernández Guijarro et al. 2019).

To qualify and strengthen such care practices, our study further analysed factors associated with obesity among users of specialized mental health services, providing evidence to guide targeted preventive and therapeutic actions within these settings. In this context, the prevalence and associated factors were investigated using multivariate Poisson regression analysis with robust variance, identifying several sociodemographic, behavioural, and clinical characteristics significantly associated with obesity in this population.

Female participants had a higher prevalence of obesity than males, consistent with findings from population‐based Brazilian studies (Ferreira et al. 2019; Sousa et al. 2021; Melo et al. 2020). This pattern has also been observed in samples of individuals with severe mental illness (Appuhamy et al. 2023) and psychiatric outpatients in Japan (Saiga et al. 2013), suggesting that gender disparities in obesity extend to populations with mental disorders. Biological differences in body composition, hormonal regulation, and social roles may contribute to these differences, as women are more likely to experience weight gain associated with psychotropic medications and lifestyle limitations imposed by psychiatric symptoms.

Regarding race/ethnicity, individuals who self‐identified as Black had a higher prevalence of obesity compared to White participants. Similar findings were reported by Ferreira et al. (2019) in the general Brazilian population, where African‐Brazilian adults exhibited greater obesity prevalence. Possible explanations include structural inequalities, differential access to health‐promoting resources, and the intersection between race, socioeconomic status, and mental health, which may amplify vulnerability to obesity. In addition, evidence from the Brazilian context suggests that experiences of racial discrimination are associated with less healthy dietary patterns, including higher consumption of ultra‐processed foods rich in sugars and fats and lower intake of fruits and vegetables, as well as behaviours such as emotional eating and loss of control over eating (Fanton et al. 2024). These factors may contribute to the increased risk of obesity in this population.

Participants with systemic arterial hypertension showed higher prevalence of obesity, a result consistent with national (Melo et al. 2020; Sousa et al. 2021) and international studies (Appuhamy et al. 2023; Saiga et al. 2013). This finding reflects the well‐established relationship between obesity and metabolic and cardiovascular comorbidities, reinforcing the need for integrated care models addressing both physical and mental health in psychiatric services.

Smoking was negatively associated with obesity in the present study, in line with results from Sousa et al. (2021) and Appuhamy et al. (2023), who also identified lower prevalence of overweight or obesity among smokers. Although nicotine is known to suppress appetite and increase metabolism, the apparent ‘protective’ effect of smoking must be interpreted cautiously, given the broader harmful health impacts of tobacco use. Nonetheless, this association suggests the need to anticipate potential weight gain during smoking cessation interventions in psychiatric populations (Golden and Davis 2025).

Users treated in substance use disorder centres (CAPS AD) and those diagnosed with substance‐related disorders exhibited a lower prevalence of obesity compared to individuals with anxiety disorders or those followed in general psychiatric outpatient clinics. This negative association may be partially explained by the metabolic consequences of chronic substance use, such as appetite suppression and nutritional deficiencies, as well as by socioeconomic and lifestyle factors influencing diet and physical activity. Similar findings were reported in the multicentre Japanese study by Ishii et al. (2024), which identified lifestyle patterns and treatment characteristics, such as the use of first‐ and second‐generation antipsychotics and frequency of eating out, as key determinants of obesity among psychiatric outpatients. Moreover, the observed difference between service types suggests that users of CAPS AD may have distinct lifestyle profiles or medication exposures compared to those in general outpatient settings. Participation in psychosocial rehabilitation and group‐based activities typical of CAPS AD services may also contribute to greater opportunities for physical activity and social engagement, which could help mitigate obesity risk in this population.

Self‐rated health was also associated with obesity, with participants perceiving their health as ‘good’ or ‘poor/very poor’ showing higher prevalence of obesity compared to those reporting ‘excellent/very good’ health. A comparable pattern was reported by Sousa et al. (2021), where individuals with worse self‐rated health were more likely to be overweight. This perception may reflect increased awareness of physical limitations or comorbidities commonly associated with obesity.

Overall, our findings are consistent with the broader literature indicating that obesity among individuals with mental disorders is multifactorial, influenced by demographic, behavioural, and clinical factors. However, some contrasts, such as the lower prevalence among those with substance‐related disorders, highlight the heterogeneity within mental health populations and the importance of context‐specific prevention strategies. The literature therefore highlights the need for integrated interventions addressing psychopharmacological management, nutrition, and lifestyle modification within mental health services, as well as the importance of educational approaches in these settings, which are fundamental for promoting behavioural change (Kengeriski et al. 2014). In addition, the study by Rosenbaum et al. (2014) demonstrated that educational interventions for nurses can also improve the assessment of cardiometabolic markers in people with mental disorders (Happell et al. 2016).

### 5.1 Strengths and Limitations of the Work

A key strength of this study is that it represents one of the few investigations conducted in an upper‐middle‐income country context that examines both the prevalence of obesity and its associated factors among users of community‐based mental health services. This contributes valuable evidence to a field where data remain scarce, particularly in settings marked by social inequities and constrained health‐system resources. The study also benefits from a robust sample and the direct measurement of anthropometric indicators, which enhances the accuracy of obesity classification and strengthens the reliability of the findings.

However, some limitations should be acknowledged. The cross‐sectional design does not allow causal inferences, limiting the ability to establish temporal relationships between obesity and associated factors. Although anthropometric data were directly measured, the study did not include other relevant behavioural or lifestyle variables, such as dietary intake and medication use (particularly antipsychotics), which could have provided a more comprehensive understanding of the mechanisms underlying obesity in this population. Additionally, because the study was conducted in a single municipality, the findings may not be generalizable to other regions or health‐system contexts.

### 5.2 Recommendations for Further Research

Future studies should incorporate longitudinal designs to examine temporal changes in weight status and identify causal pathways linking mental health conditions, service use, and obesity. Research should also integrate behavioural and environmental variables, such as physical activity, dietary patterns, medication use (particularly antipsychotics), and food insecurity. Mixed‐methods approaches may help capture the influence of social determinants, stigma, and service organization on physical health outcomes. Importantly, implementation research is needed to test strategies that strengthen routine screening, promote interprofessional collaboration, and integrate physical and mental healthcare within community‐based services. Future research should also consider the limitations associated with the use of self‐reported measures, particularly regarding potential reporting biases that may affect the accuracy of the findings.

## 6 Conclusion

This study demonstrates a substantial burden of obesity and metabolic risk among users of specialized mental health services, surpassing national and international estimates for the general population and underscoring the growing epidemiological importance of physical health conditions within the Brazilian Psychosocial Care Network. By identifying demographic, behavioural, and clinical factors significantly associated with obesity, such as female sex, Black race/ethnicity, hypertension, smoking status, and type of service and diagnosis, our findings highlight the multifactorial nature of obesity in mental health populations and the heterogeneity of risk profiles across different service settings.

The high prevalence of central adiposity and the evident gaps in routine anthropometric monitoring reveal missed opportunities for early detection, prevention, and integrated care within community‐based services. These results reinforce the urgent need to incorporate systematic physical health assessments into mental health routines, to strengthen the roles of nursing and interdisciplinary teams, and to improve coordination between psychosocial care services and primary healthcare.

Taken together, these findings offer practical directions for developing targeted interventions to address metabolic health within psychiatric care, particularly by promoting physical activity, supporting healthier dietary practices, and incorporating integrated lifestyle approaches. At the same time, they highlight the influence of broader structural determinants, including socioeconomic inequities and racialized vulnerabilities, in shaping obesity risk among people with mental disorders. Moving forward, advancing integrated, equity‐oriented, and evidence‐informed care models will be essential to improving health outcomes and reducing preventable morbidity in this population.

## 7 Relevance to Clinical Practice

The findings from this study demonstrate that obesity and metabolic risk should be addressed as integral components of care within community mental health services. At the practice level, integrating routine weight and height measurement, nutritional counselling, and structured referral pathways with primary care may reduce care fragmentation and improve health outcomes. These practices can strengthen nursing and multidisciplinary care through early risk identification, health education, and coordinated, equity‐sensitive approaches that address social and racial disparities.

## Author Contributions

All listed authors meet the authorship criteria in accordance with the most recent guidelines of the International Committee of Medical Journal Editors (ICMJE), and all authors have reviewed and approved the final version of the manuscript. Conceptualization: Carlos Treichel. Methodology: Carlos Treichel. Data curation: Thaiane Diomedece, Bruno Silva, Ana Lourencetti, Carlos Treichel, Mark Costa, Maria O'Connell, Chyrell Bellamy. Formal analysis: Thaiane Diomedece, Carlos Treichel. Investigation: Carlos Treichel. Writing – original draft: Thaiane Diomedece, Bruno Silva, Ana Lourencetti, Carlos Treichel, Mark Costa, Maria O'Connell, Chyrell Bellamy. Writing – review and editing: Thaiane Diomedece, Bruno Silva, Ana Lourencetti, Carlos Treichel, Mark Costa, Maria O'Connell, Chyrell Bellamy. Visualization: Carlos Treichel. Supervision: Carlos Treichel. Project administration: Carlos Treichel, Ana Lourencetti.

## Funding

This work was supported by the Fundação de Amparo à Pesquisa do Estado de São Paulo (FAPESP), grant #2023/18317‐2, Coordenação de Aperfeiçoamento de Pessoal de Nível Superior ‐ Brasil (CAPES), grant #001.

## Ethics Statement

This study was approved by the accredited Research Ethics Committee (CAAE 70316723.0.0000.5392). All procedures were conducted in accordance with national standards and the Declaration of Helsinki.

## Consent

All participants provided free and informed written consent before data collection.

## Conflicts of Interest

The authors declare no conflicts of interest.

## Data Availability

The data that support the findings of this study are available from the corresponding author upon reasonable request.
